# p21/WAF1 expression as related to p53, cell proliferation and prognosis in epithelial ovarian cancer

**DOI:** 10.1038/sj.bjc.6690298

**Published:** 1999-04

**Authors:** M A Anttila, V-M Kosma, J Hongxiu, J Puolakka, M Juhola, S Saarikoski, K Syrjänen

**Affiliations:** Department of Obstetrics and Gynecology, Kuopio University Hospital, Kuopio, Finland; Department of Pathology, Kuopio University Hospital, Kuopio, Finland; Department of Pathology and Forensic Medicine, University of Kuopio, Kuopio, Finland; Department of Pathology, Johns Hopkins Hospital, Baltimore, USA; Department of Obstetrics and Gynecology, Jyväskylä Central Hospital, Jyväskylä, Finland; Department of Pathology, Jyväskylä Central Hospital, Jyväskylä, Finland

**Keywords:** p21/WAF1, p53, Ki-67, epithelial ovarian cancer, prognosis

## Abstract

The role and prognostic value of the tumour suppressor p21/WAF1 expression in epithelial ovarian cancer has not yet been defined. Therefore, the expression of p21/WAF1 was assessed immunohistochemically (IHC) in 316 epithelial ovarian malignancies in relation to p53, cell proliferation and patient survival. p21/WAF1 expression was inversely correlated with p53 and cell proliferation. Low p21/WAF1 expression was significantly associated with high grade of the tumour (*P* = 0.0005), advanced FIGO stage (*P* = 0.001) and primary residual tumour (*P* = 0.0001). Low p21/WAF1 expression was a marker of poor overall survival (*P* = 0.012). Similarly, p53-positivity and high cell proliferative activity were significant predictors of poor survival in univariate analyses. Moreover, the patients with p21–/p53+ tumours had a poorer overall (*P* < 0.00005) and recurrence-free (*P* = 0.0005) survival in univariate analyses, and the p21/p53 expression independently predicted tumour recurrence in Cox's multivariate analysis. Our results suggest that p21/WAF1 expression is mostly p53-dependent in epithelial ovarian cancer. High p21/WAF1 expression seems to function as a negative cell cycle regulator and as a marker of favourable disease outcome in epithelial ovarian cancer. In addition, the patients with their tumour expressing no or low p21/WAF1 protein but positive for p53 had a notably higher risk of recurrent disease, implicating that these patients might be more prone to treatment failures. © 1999 Cancer Research Campaign


*British Journal of Cancer* (1999) **79**(11/12), 1870–1878
© 1999 Cancer Research Campaign
Article no. bjoc.1998.0298

### p21/WAF1 expression as related to p53, cell proliferation and prognosis in epithelial ovarian cancer

**MA Anttila[1,3], V-M Kosma[2,3], J Hongxiu[4], J Puolakka[5], M Juhola[6], S Saarikoski[1] and K Syrjänen[2,3]**

[1]Department of Obstetrics and Gynecology, Kuopio University Hospital, Kuopio, Finland; [2]Department of Pathology, Kuopio University Hospital, Kuopio, Finland; [3]Department of Pathology and Forensic Medicine, University of Kuopio, Kuopio, Finland; [4]Department of Pathology, Johns Hopkins Hospital, Baltimore, USA; [5]Department of Obstetrics and Gynecology, Jyväskylä Central Hospital, Jyväskylä, Finland and [6]Department of Pathology, Jyväskylä Central Hospital, Jyväskylä, Finland

**Summary** The role and prognostic value of the tumour suppressor p21/WAF1 expression in epithelial ovarian cancer has not yet been defined. Therefore, the expression of p21/WAF1 was assessed immunohistochemically (IHC) in 316 epithelial ovarian malignancies in relation to p53, cell proliferation and patient survival. p21/WAF1 expression was inversely correlated with p53 and cell proliferation. Low p21/WAF1 expression was significantly associated with high grade of the tumour ($P = 0.0005$), advanced FIGO stage ($P = 0.001$) and primary residual tumour ($P = 0.0001$). Low p21/WAF1 expression was a marker of poor overall survival ($P = 0.012$). Similarly, p53-positivity and high cell proliferative activity were significant predictors of poor survival in univariate analyses. Moreover, the patients with p21–/p53+ tumours had a poorer overall ($P < 0.00005$) and recurrence-free ($P = 0.0005$) survival in univariate analyses, and the p21/p53 expression independently predicted tumour recurrence in Cox's multivariate analysis. Our results suggest that p21/WAF1 expression is mostly p53-dependent in epithelial ovarian cancer. High p21/WAF1 expression seems to function as a negative cell cycle regulator and as a marker of favourable disease outcome in epithelial ovarian cancer. In addition, the patients with their tumour expressing no or low p21/WAF1 protein but positive for p53 had a notably higher risk of recurrent disease, implicating that these patients might be more prone to treatment failures.

**Keywords:** p21/WAF1; p53; Ki-67; epithelial ovarian cancer; prognosis

As a result of DNA damage, p53 induces the expression of several downstream genes including p21/WAF1 (El-Deiry et al, 1994). The p21 protein binds to and inhibits cyclin-dependent kinases (Cdks) resulting in cell-cycle arrest during the G1 to S transition (Harper et al, 1993). The expression of p21/WAF1 has been shown to correlate inversely with a variety of cell proliferation markers (El-Deiry et al, 1993; Doglioni et al, 1996; Mateo et al, 1997). In addition, p21 immunoreactivity was recently observed to be related to cell differentiation as well (El-Deiry et al, 1995). Furthermore, the lack of p21 immunoreactivity has been related to other prognostic factors and survival in breast (Wakasugi et al, 1997) and gastric cancers (Gomyo et al, 1997; Ogawa et al, 1997) but no data on the prognostic significance of p21/WAF1 expression in epithelial ovarian cancer are available as yet.

In Finland 595 ovarian cancers were diagnosed in 1995 and the mortality rate was 6.6/100 000 (Finnish Cancer Registry, 1997). In Western countries the incidence rate of ovarian cancer is about 15 per 100 000 women (Boring et al, 1994; Engeland et al, 1995) and in the United States it is estimated that 26 800 women will manifest ovarian cancer, and of these there will be 14 200 deaths in 1997 (Parker et al, 1997). Previous studies have shown that the p53 gene is mutated in 30–80% of the ovarian cancers and these genetic changes closely correlate with the accumulation of mutant p53 in tumour tissue (Marks et al, 1991; Kohler et al, 1993*a*; Kohler et al, 1993*b*). p21/WAF1 protein expression has been detected in the cells with wild-type p53, but not in those lacking p53 protein activity (El-Deiry et al, 1994). Inferred from these observations, dysregulation of p21/WAF1 could provide growth advantage to tumour cells also in ovarian cancer. The p21/WAF1 gene is located on chromosome 6p21.2, and ovarian tumours frequently show losses of heterozygosity in this region (Wan et al, 1994). Moreover, some recent data indicate that p53-independent pathways may also lead to an increase in p21/WAF1 expression (Jiang et al, 1994; Michieli et al, 1994). In epithelial ovarian cancer, no correlation between p21/WAF1 expression and p53 or Ki-67 has been demonstrated so far (Barboule et al, 1995, Werness et al, 1997).

We used immunohistochemistry (IHC) to assess the expression of p21/WAF1, p53 and the cell proliferation marker Ki-67 in a cohort of 316 patients with epithelial ovarian cancer. In addition to exploring the inter-relationships of these cell-cycle proteins, our major aim was to analyse whether p21/WAF1 protein levels are related to significant clinical data, histological parameters and disease outcome of this group of malignancies.

### MATERIALS AND METHODS

### Clinical data

The material of the present study was selected from a consecutive series of 445 women diagnosed and treated for ovarian malignancy at Kuopio University Hospital and Jyväskylä Central Hospital, Finland, between 1976 and 1992 (and subsequently followed-up until September 1996), by excluding the nonepithelial type of neoplasia from the present study. Patients who were given any treatment before the primary operation were also excluded.

Accepted 29 September 1998
*Received 18 June 1998*
*Revised 9 September 1998*

*Correspendence to:* MA Anttila

**Table 1** Clinical data of the patients

| | | |
|---|---|---|
| **Age (years)** | | |
| **Median** | **62** | |
| **Range** | **18–85** | |
| Histological type | | |
| Epithelial | | |
| Serous | 111 | (35%) |
| Mucinous | 36 | (11%) |
| Endometrioid | 84 | (27%) |
| Clear cell | 32 | (10%) |
| Miscellaneous[a] | 53 | (17%) |
| Histological grade | | |
| 1 | 48 | (15%) |
| 2 | 107 | (34%) |
| 3 | 161 | (51%) |
| FIGO stage | | |
| I | 86 | (27%) |
| II | 47 | (15%) |
| III | 150 | (48%) |
| IV | 33 | (10%) |
| Primary residual tumour | | |
| No data | 30 | (10%) |
| None | 121 | (38%) |
| ≤ 2 cm | 53 | (17%) |
| > 2 cm | 112 | (35%) |
| Adjuvant chemotherapy | | |
| Platinum-containing | 167 | (53%) |
| No platinum-containing | 99 | (32%) |
| None | 47 | (15%) |
| Chemotherapy response | | |
| No data | 7 | (2%) |
| CR | 142 | (45%) |
| PR | 40 | (13%) |
| SD | 22 | (7%) |
| PD | 58 | (18%) |
| Cause of death | | |
| Ovarian cancer | 204 | (64%) |
| Other cause | 34 | (11%) |
| Alive | 78 | (25%) |
| Total | 316 | (100%) |

[a]Includes 20 mixed epithelial, one Brenner, 32 unclassified epithelial. PR, partial response; CR, complete response; SD, stable disease; PD, progressing disease, no chemotherapy 47 (15%).

Depending on the availability of representative tumour material, 316 valid immunostainings for p53 and Ki-67 and 305 for p21/WAF1, respectively, could be completed in the present study.

All tumours were staged according to the International Federation of Gynecology and Obstetrics standards (Cancer Committee of the International Federation of Gynecology and Obstetrics, 1989). Retrospective review of the patient files was performed to obtain all pertinent data on the primary tumour, type of surgery, adjuvant treatment, recurrence and survival. In case of incomplete primary information, the patients were retrospectively assigned a FIGO staging on the basis of these patient files. Patients who died because of any postoperative complications were excluded from the survival analyses. The pertinent clinicopathological data of the patients are summarized in Table 1.

Of the 316 patients, 121 underwent radical surgery (i.e. no primary residual tumour), 230 received postoperative chemotherapy, nine received postoperative radiotherapy and 39 women received both therapies. Second-look or other debulking operation was performed on 29% of the patients. Disease recurrence was observed in 76 patients (44%) and no recurrence in 95 patients (56%). The median follow-up time for all patients ($n$ = 316) was 29 months (range 1–237 months) and for patients still alive ($n$ = 78), 108 months (range 22–237 months).

### Histology

The primary tumour samples were fixed in 10% formalin and embedded in paraffin. From each sample, 5-μm thick sections were stained with haematoxylin and eosin (HE). Histological typing and grading were done according to the WHO classification (Serov et al, 1973). All tumours were graded as either well, moderately or poorly differentiated by one pathologist (KS). Borderline tumours were excluded from the study. This microscopic re-evaluation was performed in the most representative slides, equivalent to the ones used for IHC analyses.

### Immunohistochemistry

### *p21/WAF1 and p53 immunostaining*

Sections were deparaffinized, rehydrated, washed for 2 × 5 min with distilled water and for p21 analysis boiled in a microwave oven in 0.01-M citrate buffer (pH = 6.0) for 5 × 5 min for antigen retrieval. Endogenous peroxidase activity was blocked by 5% hydrogen peroxide for 5 min, followed by washings for 2 × 3 min with distilled water and 2 × 5 min with PBS (pH = 7.2). After blocking the nonspecific staining with normal horse serum, the tissue sections were incubated with the p21-specific mouse monoclonal antibody (NCL-WAF-1, Novocastra Laboratories Ltd, UK) at a working dilution of 1:10, overnight at + 4 °C. Samples were washed twice for 5 min with PBS and incubated for 30 min with biotinylated secondary antibody (Vectastain ABC Elite Kit, Vector Laboratories, CA, USA) in PBS. After two washings for 5 min in PBS, the sections were incubated for 40 min in preformed avidin-biotinylated peroxidase complex solution. Samples were washed 2 × 5 min with PBS, developed with diaminobenzidine tetrahydrochloride (DAB) substrate (Sigma, UK) for 5 min, slightly counterstained with Mayer's haematoxylin, dehydrated, cleared and mounted with DePex (BDH Limited, Poole, UK).

The p53 protein was demonstrated using a similar IHC staining protocol. We used a polyclonal CM1 (Novocastra Laboratories Ltd, Newcastle upon Tyne, UK) at a working dilution of 1:1200 without antigen retrieval. In each batch, known p53- and p21-positive tumour samples were used as positive controls, and the same biopsy processed without the primary antibody was used as a negative control.

### *Ki-67 immunostaining*

For Ki-67 detection, the monoclonal anti-Ki-67 antibody (MIB1, Immunotech, France) was used and diluted at 1:50 in PBS. In brief, 5-μm sections from the tumours were deparaffinized, rehydrated, and washed for 5 min with phosphate-buffered saline (PBS). The sections were heated in a microwave oven for 5 × 5 min in 0.01-M citrate buffer (pH = 6.0). After microwave, the slides were rinsed with Tris-buffered saline (pH = 7.4). Endogenous peroxidase activity was blocked by 5% hydrogen peroxide for 5 min, followed by a wash for 5 min with PBS. Indirect ABC technique was used as described above. Tonsillar tissue sections served as positive controls.

© Cancer Research Campaign 1999 

**Table 2** The correlation of p21/WAF1 expression with p53 and cell proliferation in epithelial ovarian cancer

| | Sample No. | p21/WAF1 Expression (%) Low | High | $\chi^2$ | df | *P* |
|---|---|---|---|---|---|---|
| p53 expression | 305 | | | 21.5 | 1 | < 0.000005 |
| Negative | 224 | 63 | 37 | | | |
| Positive | 81 | 90 | 10 | | | |
| Cell proliferation | 303 | | | 10.3 | 1 | 0.001 |
| Low | 84 | 56 | 44 | | | |
| High | 219 | 75 | 25 | | | |

### *Scoring of the p21/WAF1 immunostaining*

All slides were evaluated with a microscope (field diameter = 490 μm) by one observer (MA), who was unaware of the clinical outcome of the patients. For p21, the positivity was assessed as the percentage of positively-stained tumour cell nuclei in the entire tumour area. For further analyses, p21/WAF1 immunoreactivity was categorized into two groups: (1) low expression if < 10% of the tumour cell nuclei were positive, and (2) high expression, if ≥ 10% of the tumour cell nuclei were positive. The 10% cut-off point was the 75th percentile of the p21/WAF1 expression.

### *Scoring of the p53 and Ki-67 immunostaining*

The p53 and Ki-67 immunostaining was quantified using a digitized image analysis system, CAS 200 (Cell Analysis System Inc., Elmhurst, IL, USA) by one observer (MA), who was unaware of the clinical outcome of the patients. A quantitative ER/PR Package (Cell Analysis System Inc, Version 2.0) for p53 and a software package (Quantitative Proliferation Index) for Ki-67 utilizing two-colour image analysis system to calculate the proportion of p53- and Ki-67-positive area in relation to the total nuclear area was used interactively. The p53- and Ki-67-positive tumour cell nuclei

**Table 3** The relationships between p21/WAF1 expression and clinicopathological factors in epithelial ovarian cancer

| Factor | Sample No. | p21/WAF1 Expression (%) Low | High | $\chi^2$ | df | *P* |
|---|---|---|---|---|---|---|
| Histological type | 305 | | | 42.8 | 4 | < 0.000005 |
| Serous | 110 | 76 | 25 | | | |
| Mucinous | 31 | 77 | 23 | | | |
| Endometrioid | 81 | 73 | 27 | | | |
| Clear cell | 31 | 19 | 81 | | | |
| Miscellaneous | 52 | 81 | 19 | | | |
| Histological grade | 305 | | | 12.1 | 1 | 0.00049 |
| 1 | 43 | 58 | 42 | | | |
| 2 | 106 | 60 | 40 | | | |
| 3 | 156 | 80 | 20 | | | |
| FIGO stage | 305 | | | 10.4 | 1 | 0.001 |
| I | 81 | 59 | 41 | | | |
| II | 46 | 57 | 43 | | | |
| III | 147 | 78 | 22 | | | |
| IV | 31 | 77 | 23 | | | |
| Primary residual tumour | 279 | | | 14.7 | 1 | 0.0001 |
| None | 118 | 60 | 40 | | | |
| ≤ 2 cm | 51 | 63 | 37 | | | |
| > 2 cm | 110 | 84 | 16 | | | |
| Chemotherapy response | 255 | | | 4.2 | 3 | 0.24 |
| CR | 136 | 65 | 35 | | | |
| PR | 40 | 80 | 20 | | | |
| SD | 21 | 71 | 29 | | | |
| PD | 58 | 74 | 26 | | | |
| Recurrence | 162 | | | 1.3 | 1 | 0.26 |
| No | 91 | 60 | 40 | | | |
| Yes | 71 | 69 | 31 | | | |
| End state | 305 | | | 5.9 | 1 | 0.015 |
| Dead | 230 | 74 | 26 | | | |
| Alive | 75 | 59 | 41 | | | |

 *© Cancer Research Campaign 1999*

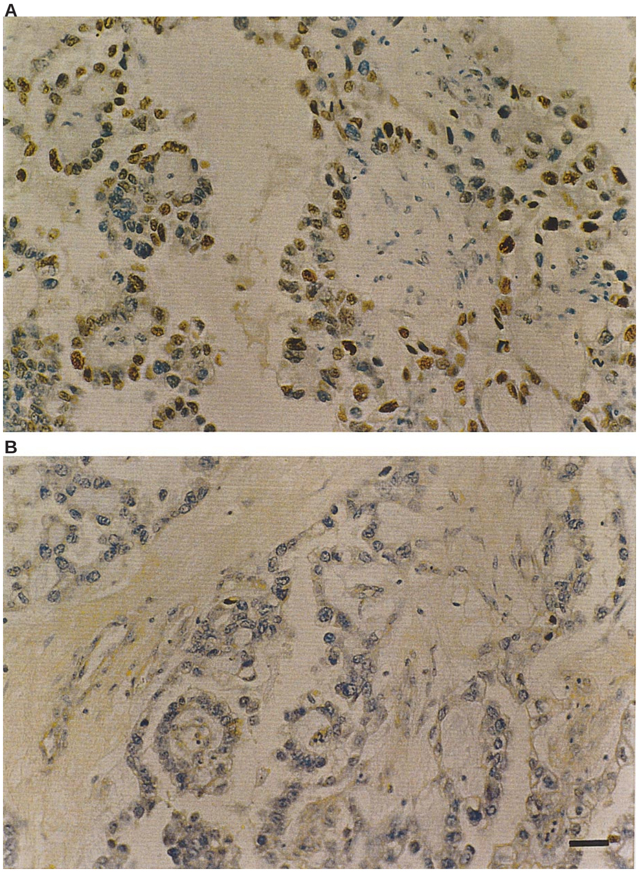

**Figure 1** Clear cell carcinoma of the ovary showing (**A**) a high expression of p21/WAF1 protein but (**B**) negative for p53 protein. Bar = 30 μm

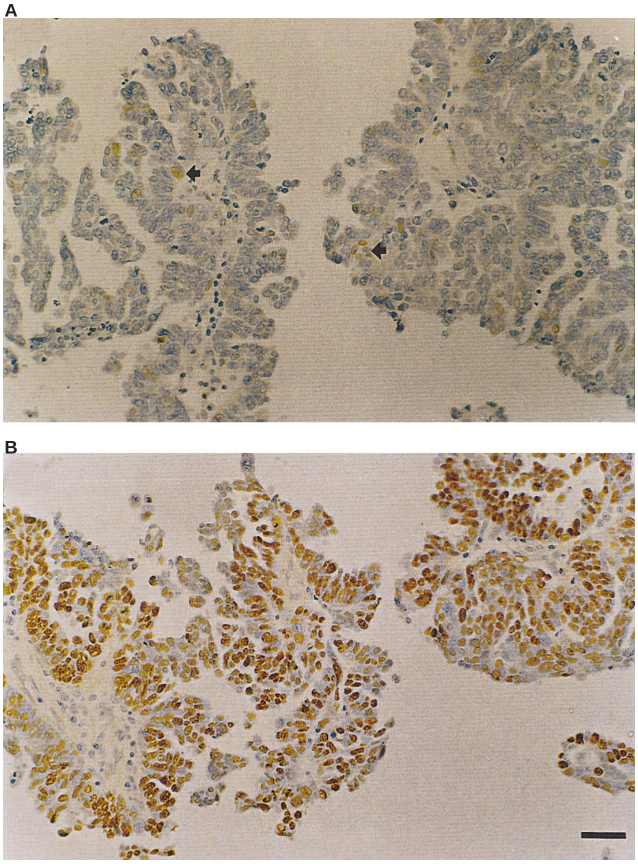

**Figure 2** Endometrioid carcinoma of the ovary showing (**A**) a few weakly p21/WAF1-positive nuclei (arrow). (**B**) Note the strong positivity for p53 protein. Bar = 50 μm as a percentage were calculated on at least 10 randomly selected fields using the × 400 magnification. For further correlation with the clinicopathological data, the p53-immunostaining was classified as (1) positive, when over 10% nuclei stained positive and (2) negative when less than 10% of the nuclei were positive (Kohler et al, 1993a). The Ki-67 positivity was classified into two groups: (1) low proliferation group when < 20% of the tumour cells were Ki-67-positive and (2) high proliferation group when ≥ 20% of the tumour cells were positive (Garzetti et al, 1995).

### Statistical analyses

The SPSS-Win 7.5 program package was used in a PC computer for basic statistical calculations. First, the relationships (Spearman correlations) between p21, p53 and Ki-67 expression levels were analysed. Each parameter in the analysis was considered as a continuous variable. The inter-relationships between the categorical IHC variables and their association with clinicopathological parameters were examined by contingency tables, which were further analysed by $\chi^2$-tests. Univariate survival analyses were based on the Kaplan–Meier method (log-rank analysis) (Kaplan and Meier, 1958). Multivariate survival analysis was done with the SPSS-Cox programme package using the log likelihood ratio significance test in forward-stepwise manner (Cox, 1972). Overall survival was defined as the time interval between the date of surgery and the date of death due to ovarian cancer. Recurrence-free survival was defined by the time interval between the date of surgery and the date of diagnosed recurrence. Probability values less than 0.05 were regarded as significant. In Cox's multivariate analysis a removal limit of $P < 0.10$ was used as an additional criteria.

### RESULTS

### p21/WAF1 immunostaining

A total of 305 primary ovarian tumours were evaluable for p21/WAF1 immunostaining. p21/WAF1 staining was confined to the tumour cell nuclei (Figure 1A). The median percentage of p21/WAF1-positive tumour cell nuclei was 3.0% (range 0–90%). Of the 305 cases, 26% ($n$ = 82) were entirely negative for p21/WAF1, whereas 30% of the tumours showed high expression

© Cancer Research Campaign 1999

**Table 4** Univariate overall and recurrence-free survival analysis of the patients

| Factor | Surviving at 5 years | | | Recurrence-free at 5 years | | |
|---|---|---|---|---|---|---|
| | *N* | % | *P*[a] | *N* | % | *P*[a] |
| Age at diagnosis (years) | 306 | | 0.0005 | 166 | | 0.15 |
| < 50 | 65 | 55 | | 40 | 68 | |
| 50–65 | 130 | 37 | | 74 | 50 | |
| > 65 | 111 | 25 | | 52 | 48 | |
| Histological grade | 306 | | 0.0002 | 166 | | 0.15 |
| 1 | 48 | 54 | | 31 | 67 | |
| 2 | 106 | 48 | | 63 | 62 | |
| 3 | 152 | 23 | | 72 | 43 | |
| Histological type | 306 | | 0.096 | 166 | | 0.0098 |
| Serous | 110 | 28 | | 52 | 39 | |
| Mucinous | 36 | 54 | | 28 | 61 | |
| Endometrioid | 82 | 36 | | 41 | 67 | |
| Clear cell | 31 | 51 | | 22 | 62 | |
| Miscellaneous | 47 | 35 | | 23 | 53 | |
| Primary residual tumour | 281 | | < 0.00005 | 158 | | < 0.00005 |
| None | 123 | 70 | | 105 | 70 | |
| ≤ 2 cm | 53 | 20 | | 30 | 38 | |
| > 2 cm | 105 | 8 | | 23 | 16 | |
| p21/WAF1 expression | 295 | | 0.012 | 157 | | 0.23 |
| Low | 203 | 31 | | 99 | 50 | |
| High | 92 | 47 | | 58 | 64 | |
| p53 expression | 306 | | < 0.00005 | 166 | | 0.0006 |
| Negative | 226 | 44 | | 137 | 60 | |
| Positive | 80 | 17 | | 29 | 28 | |
| p21/p53 expression | 295 | | < 0.00005 | 157 | | 0.0004 |
| p21–/p53+ | 70 | 15 | | 24 | 21 | |
| p21+/p53– | 84 | 47 | | 54 | 62 | |
| p21+/p53+ | 8 | 38 | | 4 | 75 | |
| p21–/p53– | 133 | 40 | | 75 | 59 | |
| Cell proliferation | 304 | | <0.00005 | 166 | | 0.002 |
| Low | 88 | 54 | | 60 | 70 | |
| High | 216 | 30 | | 106 | 46 | |

[a]Log-rank analysis.

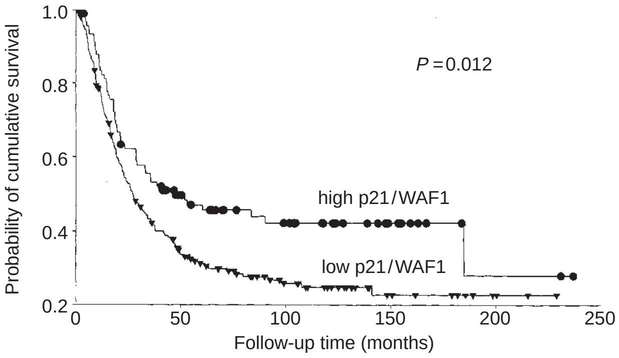

**Figure 3** Kaplan–Meier overall survival analysis of the patients with tumours expressing high or low p21/WAF1 protein

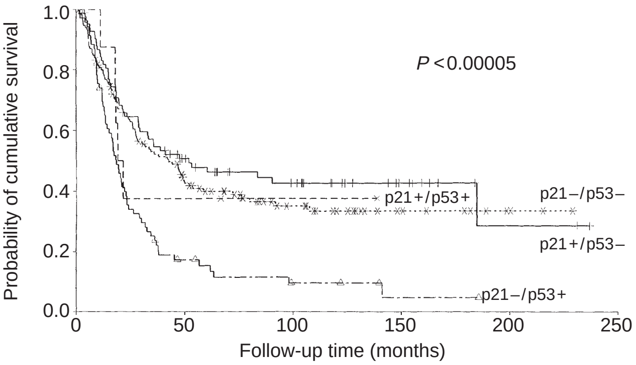

**Figure 4** Kaplan–Meier overall survival analysis of the patients with different combinations of p21/WAF1 and p53 expression of p21/WAF1, the staining being diffuse in 73% of these positive tumours.

### p53 and Ki-67 immunostaining p53 staining was confined to tumour cell nuclei (Figure 2B), and only a few cases with cytoplasmic staining were observed. Any degree of nuclear p53 immunopositivity was observed in 30% of the primary tumours. According to the 10% cut-off point, 26% ($n = 83$) of the tumours were positive for p53 protein. Similarly, the Ki-67 staining was also observed in tumour cell nuclei. The median percentage of Ki-67-positive tumour cell nuclei was 29% (range 0–78%). Accordingly, 71% of the primary tumours were considered to be highly proliferative.

© Cancer Research Campaign 1999

### Relation of p21/WAF1 expression to p53 and cell proliferation

An inverse correlation was observed between p21/WAF1 and p53 expression (Spearman, $r = -0.24$, $P < 0.0005$) (see Figures 1 and 2) and Ki-67 expression (Spearman, $r = -0.20$, $P < 0.0005$) as shown in the contingency tables (Table 2). Positive p53 expression was directly associated with high cell proliferation ($\chi^2$, $P = 0.0001$); 88% of the p53-positive tumours were highly proliferative.

### Relation of p21/WAF1 expression to the clinicopathological data

The low expression of p21/WAF1 protein was significantly associated with the high grade, advanced stage, serous and miscellaneous epithelial type of tumour (Table 3). Clear cell tumours showed high expression of p21/WAF1 protein. In those patients with the primary residual tumour > 2 cm, 84% of the tumours expressed low p21/WAF1 protein (Table 3). Importantly, 79% of the patients who eventually died of their disease during the follow-up, had low p21/WAF1 expression in their tumours.

### Relation of p53 expression and cell proliferation to the clinicopathological data

The p53-positivity was significantly associated with high grade of the tumour ($\chi^2$, $P = 0.0004$), with advanced FIGO stage ($P = 0.0001$), presence of residual tumour ($P = 0.00001$), serous histological type ($P = 0.005$) and recurrence of the disease ($P = 0.007$). A significant correlation was observed between the fatal disease outcome and p53-positivity ($P = 0.00002$). In the complete chemotherapy response group, there were more p53-negative (83%) than p53-positive tumours (18%) ($P = 0.001$).

**Table 5** Results of Cox's multivariate analysis for overall and recurrence-free survival

| Category | Beta (SE) | Relative risk (95% CI) | *P*-value |
|---|---|---|---|
| *Overall survival* | | | |
| FIGO stage | | | 0.0006 |
| I | | | |
| II | 0.51 (0.35) | 1.66 (0.84–3.28) | 0.14 |
| III | 0.88 (0.37) | 2.42 (1.17–5.01) | 0.018 |
| IV | 1.63 (0.42) | 5.12 (2.23–11.74) | 0.0001 |
| Age at diagnosis (years) | | | 0.02 |
| <50 | | | |
| 50–65 | 0.44 (0.24) | 1.55 (0.97–2.46) | 0.067 |
| >65 | 0.67 (0.24) | 1.95 (1.22–3.13) | 0.0055 |
| Primary residual tumour | | | < 0.00005 |
| None | | | |
| ≤ 2 cm | 1.17 (0.31) | 3.21 (1.73–5.94) | 0.0002 |
| > 2 cm | 1.54 (0.31) | 4.65 (2.52–8.59) | < 0.00005 |
| Adjuvant chemotherapy | | | 0.019 |
| Platinum-containing | | | |
| Non-platinum | 0.35 (0.18) | 1.42 (1.00–2.02) | 0.047 |
| None | 0.78 (0.31) | 2.19 (1.18–4.06) | 0.013 |
| *Recurrence-free survival* | | | |
| Histological type | | | 0.038 |
| Serous | | | |
| Mucinous | – 0.73 (0.46) | 0.48 (0.20–1.18) | 0.11 |
| Endometrioid | – 0.86 (0.37) | 0.42 (0.21–0.87) | 0.019 |
| Clear cell | – 0.42 (0.40) | 0.66 (0.30–1.46) | 0.31 |
| Miscellaneous | – 0.93 (0.38) | 0.39 (0.19–0.84) | 0.015 |
| Primary residual tumour | | | < 0.00005 |
| None | | | |
| ≤ 2 cm | 0.89 (0.32) | 2.43 (1.31–4.50) | 0.0049 |
| > 2 cm | 1.56 (0.32) | 4.78 (2.54–8.98) | < 0.00005 |
| p53 expression | | | |
| Negative | | | |
| Positive | 0.75 (0.30) | 2.11 (1.17–3.81) | 0.013 |
| p21/p53 expression | | | 0.0072 |
| p21–/p53+ | | | |
| p21+/p53– | – 1.06 (0.36) | 0.34 (0.17–0.70) | 0.0037 |
| p21+/p53+ | –[a] | | |
| p21–/p53– | – 1.08 (0.33) | 0.34 (0.17–0.65) | 0.0012 |

[a]Not stable due to few cases.

© Cancer Research Campaign 1999

High proliferative activity (determined by Ki-67 expression) was significantly related to the high grade ($\chi^2$, $P = 0.001$) and to the advanced stage ($P < 0.001$) of the tumour, as well as disease recurrence ($P = 0.013$) and death ($P < 0.001$).

### Survival

### *Clinicopathological factors and survival*

At the end of the follow-up 238 (75%) patients were dead, 204 (86%) due to their ovarian cancer. The median overall survival of the patients ($n = 316$) was 33 months (95% CI, 26–40) and the 5-year overall survival rate was 37%. In univariate survival analysis, the high grade disease, advanced stage, older age at diagnosis, primary residual tumour > 2 cm and platinum-containing adjuvant chemotherapy ($P = 0.046$) were significant predictors of poor overall survival (Table 4). In univariate recurrence-free analysis, only the serous histological type and the primary residual tumour > 2 cm were associated significantly with the recurrent disease (Table 4).

### *p21/WAF1 expression and survival*

The low p21/WAF1 expression was a significant predictor of poor overall survival ($P = 0.012$) in the univariate analysis (Table 4, Figure 3). The p21/WAF1 expression had no significant association with the overall survival in the different subgroups of histological grade, FIGO stage, primary residual tumour, cell proliferation or p53 expression. In the serous tumours, the high p21/WAF1 expression predicted better 5-year survival (44% vs 22%) ($P = 0.01$). In the older age group (> 65 years), the low p21/WAF1 expression predicted poor overall survival ($P = 0.04$). In the recurrence-free survival analysis, p21/WAF1 expression had no predictive value (Table 4).

### *p53 expression, proliferative activity and survival*

The p53-positivity and high proliferative activity were strongly associated with both the overall and recurrence-free survival (Table 4). Patients with p53-positive tumours had only 17% 5-year overall survival as, compared with 44% survival of the patients with p53-negative tumours ($P < 0.00005$). Similarly, highly proliferative tumours had 30% 5-year overall survival compared with that (54%) of the tumours with low proliferative activity. By combining the results of p21/WAF1 and p53 expression, the patients with p21–/p53+ tumours had a poorer prognosis in the univariate overall ($P < 0.00005$) (Fig. 4) and recurrence-free ($P = 0.0005$) survival analyses compared with patients who had other combinations of these two proteins in their tumours (Table 4). This was independent of the dichotomized histological grade (grade 1–2 vs grade 3) but not of the FIGO stage, although the prognostic value of p21–/p53+ expression reached a borderline statistical significance in patients with FIGO stages III–IV ($P = 0.05$). High cell proliferation was also a significant predictor of poor overall survival in patients with both a low ($P = 0.007$) and a high ($P = 0.03$) p21/WAF1 expression.

### *Multivariate survival analysis*

The complete data for a multivariate overall analysis was available from 265 patients. The histological grade and type, tumour stage, primary residual tumour, adjuvant chemotherapy, age at diagnosis, p21/WAF1 and p53 expression and tumour proliferation were entered into Cox's analysis. The FIGO stage, age at diagnosis, primary residual tumour and adjuvant chemotherapy proved to be significant predictors of the overall survival (Table 5). p53 expression, primary residual tumour and the histological type were significant prognostic factors of recurrence-free survival ($n = 148$) (Table 5). When the different combinations of p21/WAF1 and p53 expression replaced the p53 expression in the Cox's analysis, they retained independent statistical significance in the multivariate recurrence-free analysis. Patients with p21+/p53– tumours had a significantly lower risk for recurrent disease than patients who had p21–/p53+ tumours (Table 5).

### DISCUSSION

The p21/WAF1 gene is induced by any DNA-damaging agents that trigger G1 arrest in cells with the wild-type p53 but not in mutant p53-containing cells (El-Deiry et al, 1994). On the other hand, studies using fibroblasts from p53 knock-out mice (Michieli et al, 1994), breast carcinoma cells (Sheikh et al, 1994), human leukaemia cells (Zhang et al, 1995) and ovarian cancer cells (Elbendary et al, 1994) have shown that p53-independent pathways for p21/WAF1 induction exist as well. Due to the paucity of data on the p21/WAF1 expression and its relation to p53, tumour proliferation and patient survival in epithelial ovarian cancer, we studied the present cohort of these ovarian neoplasms by IHC.

In the present series, p21/WAF1 expression was entirely absent in 26% of the tumours. Any degree of p21/WAF1 positivity was observed in 74% of the tumours, and intense positive signals in 30% of the cases. These figures are consistent with those reported in a few previous studies, where p21/WAF1 expression was detected in 7–75% of the ovarian tumours (Barboule et al, 1995; Auer et al, 1996; Lukas et al, 1997; Werness et al 1997). Shigemasa et al (1997) studied the p21/WAF1 mRNA expression in 13 ovarian tumours and they found p53 mutation in 69% of tumours underexpressing p21 mRNA. We found a statistically significant inverse correlation between p21/WAF1 expression and p53, which is in accordance with the data of Elbendary et al (1996) in epithelial ovarian cancer cells, as well as with the observations in breast cancer (Bukholm et al, 1997). Previous studies on gynaecological malignancies have shown either no correlation between p21/WAF1 expression and p53, e.g. in ovarian (Werness et al., 1997) and endometrial cancer (Backe et al, 1997; Ito et al, 1997), or p53-independent expression of p21/WAF1 in cervival cancer (Werness et al, 1997). Our results suggest, that p21/WAF1 expression is regulated p53-dependently in the vast majority of epithelial ovarian cancers. It can also be assumed, that IHC detection of p53 accumulation is related to p53 mutation or to stabilized p53 after functional inactivation by nonmutational events (Harada et al, 1997). However, the relationship between p21/WAF1 expression and p53 is highly complex and seems to be tumour type-specific as well.

p21/WAF1 negatively regulates cell proliferation by inhibiting Cdks in some normal tissues (El-Deiry et al, 1995) and tumour cells (El-Deiry et al, 1993). The present demonstration of an inverse correlation between p21/WAF1 expression and a proliferation marker Ki-67 is in alignment with this notion (El-Deiry et al, 1993, 1995). Similarly, an inverse correlation between p21/WAF1 expression and Ki-67 was documented in endometrial cancer (Palazzo et al, 1997). On the other hand, earlier studies with ovarian (Barboule et al, 1995; Werness et al, 1997), endometrial (Backe et al, 1997) and breast cancers (Diab et al, 1997) have found no correlation between p21/WAF1 expression and tumour

 © Cancer Research Campaign 1999

proliferation. However, these data might be skewed by the limited number of cases studied. It may well be that larger series of tumours like the one of our present study are needed to demonstrate the growth-inhibitory effects of p21/WAF1. It sounds feasible that the blocking of cell proliferation by p21/WAF1 may permit cell differentiation and senescence, as shown in several human tissues, e.g. in lung carcinomas p21/WAF1 overexpression is related to histological differentiation (Marchetti et al, 1996).

The low p21/WAF1 expression was significantly associated with high grade of the tumour, advanced FIGO stage and serous or miscellaneous epithelial histological type. Accordingly, 53% of the serous and 87% of the miscellaneous epithelial tumours were poorly differentiated and the low expression of p21/WAF1 in these tumours indicates the relation of p21/WAF1 to tumour differentiation (El-Deiry et al, 1995; Doglioni et al, 1996). In concordance of the study of Werness et al (1997), we noticed a higher p21/WAF1 expression in clear cell tumours. There was a tendency for higher p21/WAF1 expression in the group with complete chemotherapy response but it did not reach statistical significance. However, these findings suggest that tumours with p21/WAF1 expression may have higher sensitivity to adjuvant chemotherapy. This is supported by the data of Kondo et al (1996), who studied glioma cells and noticed that p21/WAF1 did not induce apoptosis but conferred the susceptibility to cisplatin.

No previous studies are available on the prognostic significance of p21/WAF1 expression in epithelial ovarian malignancies. In the present study, the lower p21/WAF1 expression was a significant predictor of poorer overall survival in the univariate survival analysis (47% vs 31% of 5-year survival rate, $P = 0.012$) but not of the recurrence-free survival in either univariate or multivariate survival analyses. This prognostic significance of p21/WAF1 expression has been reported in some neoplasias (Gomyo et al, 1997; Ito et al, 1997; Jiang et al, 1997; Ogawa et al, 1997; Wagasuki et al, 1997), whereas other authors have failed to establish any such prognostic value (Ito et al, 1996; Backe et al, 1997; Diab et al, 1997). The mechanisms by which the expression of p21/WAF1 is associated with better prognosis is not known in any detail as yet. p21/WAF1 may mediate growth arrest by inhibiting further DNA synthesis, thus allowing the cells to continue their differentiation. That is substantiated by our present observation of an inverse correlation between p21/WAF1 and Ki-67, and the significant association between the p21/WAF1 upregulation and tumour differentiation ($P = 0.00049$). Furthermore, the p21/WAF1 expression seems to be related to the FIGO stage of the tumour, in that the lower p21/WAF expression is confined to advanced stage ($P = 0.001$). The same was observed in the study of Ogawa et al (1997) in gastric cancer; patients with p21/WAF1-negative tumours usually had a metastatic disease. The loss of independent predictive value of p21/WAF in the multivariate survival analyses may be explained by its p53-dependency. Thus, p53 expression seems to be a stronger independent prognostic marker of survival than p21/WAF1, masking the effect of the latter in the multivariate analysis.

Both p53 expression and tumour cell proliferative activity were of prognostic significance in the univariate overall and recurrence-free survival analyses in this study. p53 expression had also independent value in the multivariate recurrence-free analysis. Similar data have been observed in other studies, where p53-positivity has significantly predicted a poor survival (Henriksen et al, 1994; Klemi et al, 1995; van der Zee et al, 1995; Herod et al, 1996). However, contradictory results have been reported as well (Marks et al, 1991; Kohler et al, 1993*a*; Levesque et al, 1995). The observed prognostic significance of Ki-67 in the present study is consonant with the data of Garzetti et al (1995). To assess further the prognostic power of p53 and p21/WAF1, we combined the p21/WAF1 expression to that of p53. Indeed, p21–/p53+ tumours had a significantly lower survival than p21+/p53– tumours. This observation retained its significance independently of the dichotomized histological grade of the tumour. In the multivariate recurrence-free analysis, combining the p21/WAF1 expression to p53 even increased the power of p53 to predict the recurrent disease. This implicates that the p21–/p53+ ovarian epithelial neoplasia might be more prone to treatment failures.

In summary, p21/WAF1 expression seems to be p53-dependent in epithelial ovarian cancer. p21/WAF1 expression also negatively regulates cell proliferation and probably regulates cell differentiation as well. In addition, the low p21/WAF1 expression was an unfavourable prognostic sign in the univariate survival analysis, although it lost its significance in the multivariate analysis.

### ACKNOWLEDGEMENTS

The skilful technical assistance of Ms Helena Kemiläinen is gratefully acknowledged. We also extend our special thanks to Ms Pirjo Halonen for her expert advice in statistical analysis. This study was supported in part by the Special Government Funding (EVO) of Kuopio University Hospital.

### REFERENCES

Auer G, Einhorn N, Nilsson B, Silfversward C and Sjovall K (1996) Biological malignancy grading in early-stage ovarian carcinoma. *Acta Oncol* **8**: 93–98

Backe J, Gassel AM, Hauber K, Krebs S, Bartek J, Caffier H, Kreipe H-H, Muller-Hermelink H-K and Dietl J (1997) p53 protein in endometrial cancer is related to proliferative activity and prognosis but not to expression of p21 protein. *Int J Gynecol Pathol* **16**: 361–368

Barboule N, Mazars P, Baldin V, Vidal S, Jozan S, Martel P and Valette A (1995) Expression of $p21^{WAF1/CIP1}$ is heterogeneous and unrelated to proliferation index in human ovarian carcinoma. *Int J Cancer* **63**: 611–615

Boring CC, Squires TS, Tong T and Montgomery S (1994) Cancer statistics, 1994. *CA Cancer J Clin* **44**: 7–26

Bukholm IK, Nesland JM, Karesen R, Jacobsen U and Borresen AL (1997) Relationship between abnormal p53 protein and failure to express p21 protein in human breast carcinomas. *J Pathol* **181**: 140–145

Cancer Committee of the International Federation of Gynecology and Obstetrics (1989) Staging Announcement: FIGO Cancer Committee. *Gynecol Oncol* **25**: 383–385

Cox DR (1972) Regression models and life tables with discussion. *J Stat Soc B* **34**: 187–192

Diab SG, Yu Y, Hilsenbeck SG, Allred DC and Elledge RM (1997) WAF1/CIP1 protein expression in human breast tumors. *Breast Cancer Res Treat* **43**: 99–103

Doglioni C, Pelosio P, Laurino L, Macri E, Meggiolaro E, Favretti F and Barbareschi M (1996) p21/WAF1/CIP1 expression in normal mucosa and in adenomas and adenocarcinomas of the colon: its relationship with differentiation. *J Pathol* **179**: 248–253

Elbendary A, Berchuck A, Davis P, Havrilesky I, Bast RC Jr, Iglehart JD and Marks JR (1994) Transforming growth factor beta 1 can induce CIP1/WAF1 expression independent of the p53 pathway in ovarian cancer cells. *Cell Growth Differ* **5**: 1301–1307

Elbendary AA, Cirisano FD, Evans AC, Davis PL, Iglehart JD, Marks JR and Berchuk A (1996) Relationship between p21 expression and mutation of p53 tumor suppressor gene in normal and malignant ovarian epithelial cells. *Clin Cancer Res* **2**: 1571–1575

El-Deiry WS, Harper JW, O'Connor PM, Velculescu VE, Canman CE, Jackman J, Pietenpol JA, Burrell M, Hill DE, Wang Y, Wiman KG, Mercer WE, Kastan MB, Kohn KW, Elledge SJ, Kinzler KW and Vogelstein B (1994) WAF1/CIP1 is induced in p53-mediated G1 arrest and apoptosis. *Cancer Res* **54**: 1169–1174

© Cancer Research Campaign 1999 

El-Deiry WS, Tokino T, Velculescu VE, Levy DB, Parsons R, Trent JM, Lin D, Mercer WE, Kinzler KW and Vogelstein B (1993) WAF1, a potential mediator of p53 tumor suppression. *Cell* **75**: 817–825

El-Deiry WS, Tokino T, Waldman T, Oliner JD, Velculescu VE, Burrell M, Hill DE, Healy E, Rees JL, Hamilton SR, Kinzler KW and Vogelstein B (1995) Topological control of $p21^{WAF1/CIP1}$ expression in normal and neoplastic tissues. *Cancer Res* **55**: 2910–2919

Engeland A, Haldorsen T, Tretli S, Hakulinen T, Horte LG, Luostarinen T, Schou G, Sigvaldason H, Storm HH and Tulinius H (1995) Prediction of cancer mortality in the Nordic countries up to the years 2000 and 2010, on the basis of relative survival analysis: a collaborative study of five Nordic cancer registries. *APMIS Suppl* **49**: 81–83

Finnish Cancer Registry (1997) Cancer incidence in Finland 1995. Cancer statistics of the National Research and Development Center for Welfare and Health. *Cancer Society of Finland* No. **58**

Garzetti GG, Ciavattini A, Gotteri G, De Nictolis M, Stramazzotti D, Lucarini G and Biagini G (1995) Ki 67 immunostaining (MIB1 monoclonal antibody) in serous ovarian tumors: index of proliferative activity with prognostic significance. *Gynecol Oncol* **56**: 169–174

Gomyo Y, Ikeda M, Osaki M, Tatebe S, Tsujitani S, Ikeguchi M, Kaibara N and Ito H (1997) Expression of p21 (waf1/cip1/sdi1), but not p53 protein, is a factor in the survival of patients with advanced gastric carcinoma. *Cancer* **79**: 2067–2072

Harada N, Gansauge S, Gansauge F, Gause H, Shimoyama S, Imaizumi T, Mattfield T, Schoenberger MH and Beger HG (1997) Nuclear accumulation of p53 correlates significantly with clinical features and inversely with the expression of the cyclin-dependent kinase inhibitor $p21^{WAF1/CIP1}$ in pancreatic cancer. *Br J Cancer* **76**: 299–305

Harper JW, Adami GR, Wei N, Keyomarsi K and Elledge SJ (1993) The p21 Cdk-interacting protein Cip1 is a potent inhibitor of G1 cyclin-dependent kinases. *Cell* **75**: 805–816

Henriksen R, Strang P, Wilander E, Backstrom T, Tribukait B and Oberg K (1994) p53 expression in epithelial ovarian neoplasms: relationship to clinical and pathological parameters, Ki-67 expression and flow cytometry. *Gynecol Oncol* **53**: 301–306

Herod JJ, Eliopoulos AG, Warwick J, Niedobitek G, Young LS and Kerr DJ (1996) The prognostic significance of Bcl-2 and p53 expression in ovarian carcinoma. *Cancer Res* **56**: 2178–2184

Ito K, Sasano H, Matsunaga G, Sato S, Yajima A, Nasim S and Garret C (1997) Correlations between p21 expression and clinicopathological findings, p53 gene and protein alterations, and survival in patients with endometrial carcinoma. *J Pathol* **183**: 318–324

Ito Y, Kobayashi T, Takeda T, Komoike Y, Wakasugi E, Tamaki Y, Tsujimoto M, Matsuura N and Monden M (1996) Expression of p21 (WAF1/CIP1) protein in clinical thyroid tissues. *Br J Cancer* **74**: 1269–1274

Jiang H, Lin J, Su ZZ, Collart FR, Huberman E and Fisher PB (1994) Induction of differentiation in human promyelocytic HL-60 leukemia cells activates p21, WAF1/CIP1, expression in the absence of p53. *Oncogene* **9**: 3397–3406

Jiang M, Shao ZM, Wu J, Lu JS, Yu LM, Yuan JD, Han QX, Shen ZZ and Fontana JA (1997) p21/waf1/cip1 and mdm-2 expression in breast carcinoma patients as related to prognosis. *Int J Cancer* **74**: 529–534

Kaplan EL and Meier P (1958) Nonparametric estimation from incomplete observations. *J Am Stat Assoc* **53**: 457–481

Klemi PJ, Pylkkänen L, Kiilholma P, Kurvinen K and Joensuu H (1995) p53 protein detected by immunohistochemistry as a prognostic factor in patients with epithelial ovarian carcinoma. *Cancer* **76**: 1201–1208

Kohler MF, Kerns BJ, Humphrey PA, Marks JR, Bast RC Jr and Berchuck A (1993*a*) Mutation and overexpression of p53 in early-stage epithelial ovarian cancer. *Obstet Gynecol* **81**: 643–650

Kohler MF, Marks JR, Wiseman RW, Jacobs IJ, Davidoff AM, Clarke Pearson DL, Soper JT, Bast RC Jr and Berchuck A (1993*b*) Spectrum of mutation and frequency of allelic deletion of the p53 gene in ovarian cancer. *J Natl Cancer Inst* **85**: 1513–1519

Kondo S, Barna BP, Kondo Y, Tanaka Y, Casey G, Liu J, Morimura T, Kaakaji R, Peterson JW, Werbel B and Barrett GH (1996) WAF1/CIP1 increases the susceptibility of p53 non-functional malignant glioma cells to cisplatin-induced apoptosis. *Oncogene* **13**: 1279–1285

Levesque MA, Katsaros D, Yu H, Zola P, Sismondi P, Giardina G and Diamandis EP (1995) Mutant p53 protein overexpression is associated with poor outcome in patients with well or moderately differentiated ovarian carcinoma. *Cancer* **75**: 1327–1338

Lukas J, Groshen S, Saffari B, Niu N, Reles A, Wen WH, Felix J, Jones LA, Hall FL and Press MF (1997) WAF1/Cip1 gene polymorphism and expression in carcinomas of the breast, ovary, and endometrium. *Am J Pathol* **150**: 167–175

Marchetti A, Doglioni C, Barbareschi M, Buttita F, Pellegrini S, Bertacca G, Chella A, Merlo G, Angeletti CA, Palma PD and Bevilacqua G (1996) p21 RNA and protein expression in non-small cell lung carcinomas: evidence of p53-independent expression and association with tumoral differentiation. *Oncogene* **12**: 1319–1324

Marks JR, Davidoff AM, Kerns BJ, Humphrey PA, Pence JC, Dodge RK, Clarke Pearson DL, Iglehart JD, Bast RC Jr and Berchuck A (1991) Overexpression and mutation of p53 in epithelial ovarian cancer. *Cancer Res* **51**: 2979–2984

Mateo MS, Saez AL, Sanchez-Beato M, Garcia P, Sanchez-Verde L, Martinez JC, Orradre JL and Piris MA (1997) Expression of $p21^{WAF1/CIP1}$ in fetal and adult tissues: simultaneous analysis with Ki67 and p53. *J Clin Pathol* **50**: 645–653

Michieli P, Chedid M, Lin D, Pierce JH, Mercer WE and Givol D (1994) Induction of WAF1/CIP1 by a p53-independent pathway. *Cancer Res* **54**: 3391–3395

Ogawa M, Maeda K, Onoda N, Chung YS and Sowa M (1997) Loss of $p21^{WAF1/CIP1}$ expression correlates with disease progression in gastric carcinoma. *Br J Cancer* **75**: 1617–1620

Palazzo JP, Mercer WE, Kovatich AJ and McHugh M (1997) Immunohistochemical localization of $p21^{WAF1/CIP1}$ in normal, hyperplastic, and neoplastic uterine tissues. *Hum Pathol* **28**: 60–66

Parker SL, Tong T, Bolden S and Wingo PA (1997) Cancer statistics, 1997. *CA Cancer J Clin* **47**: 5–27

Serov SF, Scully R and Sobin LH (1973) Histological typing of ovarian tumours. *International Histological Classification of Tumors*, no. **9**. Geneva: WHO

Sheikh MS, Li XS, Chen JC, Shao ZM, Ordonez JV and Fontana JA (1994) Mechanisms of regulation of WAF1/Cip1 gene expression in human breast carcinoma: role of p53-dependent and independent signal transduction pathways. *Oncogene* **9**: 3407–3415

Shigemasa K, Hu C, West CM, Moon SH, Parham GP, Parmley TH, Korourian S, Baker VV and O'Brien TJ (1997) p21: a monitor of p53 dysfunction in ovarian neoplasia. *Int J Gynecol Cancer* **7**: 296–303

Wakasugi E, Kobayashi T, Tamaki Y, Ito Y, Miyashiro I, Komoike Y, Takeda T, Shin E, Takatsuka Y, Kikkawa N, Monden T and Monden M (1997) p21 (Waf1/Cip1) and p53 protein expression in breast cancer. *Am J Clin Pathol* **107**: 684–691

van der Zee AG, Hollema H, Suurmeijer AJ, Krans M, Sluiter WJ, Willemse PH, Aalders JG and de Vries EG (1995) Value of P-glycoprotein, glutathione S-transferase pi, c-erbB-2, and p53 as prognostic factors in ovarian carcinomas. *J Clin Oncol* **13**: 70–78

Wan M, Zweizig S, D'Ablaing G, Zheng J, Velicescu M and Dubeau L (1994) Three distinct regions of chromosome 6 are targets of loss of heterozygosity in human ovarian carcinomas. *Int J Oncol* **5**: 1043–1048

Werness BA, Jobe JS, DiCioccio RA and Piver MS (1997*a*) Expression of the p53 induced tumor suppressor $p21^{waf1/cip1}$ in ovarian carcinomas: correlation with p53 and Ki-67 immunohistochemistry. *Int J Gyn Pathol* **16**: 149–155

Werness BA, Wang H-Q, Chance J and Goldstein DJ (1997*b*) p53-independent expression of $p21^{waf1/cip1}$ in preinvasive and invasive squamous neoplasms of the uterine cervix. *Mod Pathol* **10**: 578–584

Zhang W, Grasso L, McClain CD, Gambel AM, Cha Y, Travali S, Deisseroth AB and Mercer WE (1995) p53-independent induction of WAF1/CIP1 in human leukemia cells is correlated with growth arrest accompanying monocyte/macrophage differentiation. *Cancer Res* **55**: 668–674

*© Cancer Research Campaign 1999*
